# Mortality in patients with diabetic foot ulcer: a retrospective study of 513 cases from a single Centre in the Northern Territory of Australia

**DOI:** 10.1186/s12902-018-0327-2

**Published:** 2019-01-03

**Authors:** Kanakamani Jeyaraman, Thomas Berhane, Mark Hamilton, Abhilash P. Chandra, Henrik Falhammar

**Affiliations:** 1 Department of Endocrinology, Royal Darwin Hospital, Darwin, Northern Territory Australia; 2 Department of Prosthetics and Orthotics, Royal Darwin Hospital, Darwin, Northern Territory Australia; 3 Department of Vascular Surgery, Royal Darwin Hospital, Darwin, Northern Territory Australia; 4 Menzies School of Health Research, Darwin, NT Australia; 5 Department of Endocrinology, Metabolism and Diabetes, Karolinska University Hospital, Stockholm, Sweden; 6 Department of Molecular Medicine and Surgery, Karolinska Institute, Stockholm, Sweden

**Keywords:** Diabetic foot ulcer, Diabetes complications, Diabetes mellitus

## Abstract

**Background:**

Diabetic foot ulcers (DFU) are a common problem in longstanding diabetes. However, mortality outcomes in Australian patients with DFU are still unclear.

**Methods:**

All patients with DFU presenting for the first time to the Multi-Disciplinary Foot Clinic (MDFC) at Royal Darwin Hospital, Northern Territory Australia, between January 2003 and June 2015 were included in this study. These patients were followed until 2017, or death. Individual patient data was extracted from hospital and primary care information systems. Kaplan-Meier survival curves were developed. The association between various risk factors and mortality was analysed using Cox regression.

**Results:**

In total 666 subjects were screened, and 513 were included in the final analysis. Of these subjects, 247 were Indigenous and 266 were non-Indigenous. The median follow-up period was 5.8 years (IQR, 3.1–9.8). The mean age at inclusion was 59.9 ± 12.3 years and 62.8% were males. The majority (93.6%) had type 2 diabetes and the median diabetes duration was 7 years (IQR, 3–12). There were 199 deaths, with a 5-year-mortality rate of 24.6%, and a 10-year-mortality rate of 45.4%. The mean age at death was 64.6 ± 11.8 years. In a multivariate analysis, the following variables were associated with mortality (adjusted HR, 95% CI): age 1.04 (1.02–1.05, *P* < 0.001); chronic kidney disease 1.22 (1.11–1.33, *P* < 0.001), and plasma albumin 0.96 (0.94–0.99, *P* < 0.05). The most common causes of death were chronic kidney disease (24.6%), cardiovascular events (19.6%), sepsis (15.6%), respiratory failure (10.0%), malignancy (9.5%) and multi-organ failure (5.0%).

**Conclusion:**

Patients with DFU have high mortality. Age, chronic kidney disease, and low albumin levels increase the risk of mortality. Strategies should focus on ulcer prevention and aggressive risk factor reduction.

## Background

Foot ulcers are a common complication of long standing diabetes mellitus (DM). Up to 25% of patients with DM develop diabetic foot ulcers (DFU) over their lifetime [1–3]. These patients have a greater than two-fold increase in mortality compared to patients with diabetes without DFU, regardless of other risk factors [2, 4]. A meta-analysis of 3619 deaths among individuals with DM reported a higher risk of all-cause mortality in patients with DFU [1]. The excess mortality was in part attributable to a greater burden of cardiovascular disease (CVD) [1]. This observation has been confirmed by recent cohort studies, which show that DFU are a significant independent predictor of mortality, even after adjusting for known CVD and other comorbidities [4, 5].

Jupiter et al. reported in their systematic review a five-year mortality rate of around 40% in patients with DFU [6]. The major risk factors for death were age, male gender, peripheral vascular disease, and renal disease. It is not known whether DFU are a surrogate marker for more advanced micro- and macro-vascular disease of DM, or if they contribute independently to mortality due to inflammatory sequelae [7]. Few studies have explored the relationship between DFU and cause-specific mortality.

The impact of DFU in Australia is large, from an individual, population, and economic perspective. In 2012, Bergin et al. reported that a limb is lost every 3 h in Australia as a result of DFU [8]. Up to 8% of all diabetes-related deaths are directly attributable to foot disease. These are especially significant in the Northern Territory (NT) of Australia, where DFUs are common, severe, and costly [9]. The NT encompasses an enormous geographical area, which is sparsely populated with limited access to health care. About 27% of all inhabitants in NT consider themselves Indigenous [10]. There are five public hospitals, and the Royal Darwin Hospital (RDH) is the only tertiary centre. More than half of patients receiving health care at RDH are Indigenous. The Australian Indigenous population has a higher burden of type 2 DM and its complications [11]. They have a higher premature mortality rate due to CVD and renal disease [12]. However, the mortality outcomes in Australian patients with DFU are still unclear.

The aims of this study were to evaluate mortality outcomes in Australian patients with DFU, and to determine the relevant factors associated with mortality and the causes of death in these patients.

## Methods

This is a retrospective study of all patients of over the age of 18-years with DFU, presenting for the first time to the Multi-Disciplinary Foot Clinic (MDFC) at RDH between January 2003 and June 2015. The MDFC consists of a team of vascular surgeons, endocrinologists, foot care nurses, diabetes nurse educators, and prosthetist/orthotists. All participating disciplines within the MDFC were involved in patient care. A structured foot-clinic proforma was used to record individual patient clinical and ulcer characteristics. Wagner’s grading system was used to classify the ulcers. Additional relevant patient data was collated from paper clinical notes and electronic medical records. Patients were reviewed regularly in the clinic, either until wound healing was achieved or death.

Peripheral neuropathy was assessed with monofilament testing. Retinopathy status was collected from optometrist or ophthalmologist reports. The definition of peripheral vascular disease (PVD) was based on the clinical diagnosis documented by the treating surgeon and, if available, by imaging such as arterial Doppler or angiography. Macrovascular disease was defined as any macrovascular complication other than PVD, including prior myocardial infarction, angioplasty, coronary artery bypass grafting, ischaemic heart disease, or stroke. Chronic kidney disease (CKD) was defined by the KDIGO criteria [13]. Patients with persistent albuminuria with a urine albumin creatinine ratio (ACR) ≥3 mg/mmol were considered to have CKD. The CKD was further staged according to the glomerular filtration rate (data not shown). Laboratory data were collected from blood tests performed within 3 months of presentation to MDFC. Laboratory measurements were done using routine assays. Data regarding culture and sensitivity were also collected. Patients were followed until death or July 2017, whichever was earlier. Cause of death was noted from death certificates (*n* = 110). For those subjects without accessible death certificates, electronic medical records were used to assign the cause of death (*n* = 67).

The study was approved by the Human Research Ethics Committee of the Northern Territory Department of Health and Menzies School of Health Research (HREC-2015-2324). Statistical analyses were performed using Stata Version 14.2. Continuous variables were expressed as mean ± SD or median (IQR), as appropriate. In order to assess differences between groups, unpaired *t*-test or Wilcoxon-Mann-Whitney U test were used for continuous variables, and Chi-2 test for categorical variables. Kaplan-Meier survival curves were generated, and the log-rank test was used to test equality of survivor functions between the various groups. Cox proportional hazards regression was used to obtain the Hazard Ratio (HR) and 95% Confidence Interval (CI) for mortality. Variables with established association with death were selected for univariate analysis, and those with a *P*-value < 0.1 were included in the multi-variate models.

## Results

Of the 666 patients screened, 153 patients were excluded (93 did not have diabetes and 60 had diabetic foot complications but no ulcer). A total of 513 patients were included in the final analysis (Table 1). The median follow-up period was 5.8 years (3.1–9.8). The mean age at first presentation was 55.9 ± 12.3 years, and 62.8% were males. The median duration of diabetes was 7 years (3–12). The majority (93.6%) had type 2 diabetes, and 53.8% were on insulin. There were almost equal numbers of Indigenous (48.1%) and non-Indigenous (51.8%) participants. In the study cohort, 30% were from remote areas of NT. A past history of DFU (57.5%) and amputations (20.3%) were common. Alcohol consumption was recorded in 63.1% of the study population, and 45.8% were current smokers. Neuropathy (90%) was the most prevalent diabetes-related complication, followed by hypertension (89%), retinopathy (49.8%), CKD (48.0%), PVD (42.6%), and macrovascular disease (33.9%).

**Table 1:** Baseline clinical and biochemical characteristics at first outpatient presentation to the multidisciplinary foot clinic

Parameter	Total cohort (*n* = 513)	Reference range
Age in years	55.9 ± 12.3	
Males	322 (62.8%)	
Remote areas	153 (29.2%)	
Indigenous ethnicity	247 (48.2%)	
Past Ulcers	295 (57.5%)	
Past amputations	104 (20.3%)	
Hypertension	456 (88.9%)	
Current Smoking	234 (45.7%)	
Non-compliance	334 (65.1%)	
Neuropathy	458 (90%)	
Retinopathy	213 (49.8%)	
PVD	216 (42.6%)	
Macrovascular disease	174 (33.9%)	
CKD	241 (47.4%)	
Insulin	278 (54.2%)	
Duration of ulcer > 4 weeks	259 (50.5%)	
Mid and hind foot ulcers	129 (25.5%)	
Ulcer area in cm2	2 (0.8–5)	
Wagner grade 3–4	53 (10.3%)	
Osteomyelitis	141 (35.6%)	
Subsequent amputation	263 (51.3%)	
Blood Haemoglobin (g/L)	120.0 ± 20.9	115–165
Blood WBC (count /L)	9.2 (7.3-11.2)	4.0–11.0
Plasma CRP (mg/L)	15.1 (8–40)	0.0–5.0
HbA1c (%)	9.1 ± 2.5	4.3–5.7
Plasma LDL (mmol/L)	2.2 ± 0.98	< 3.0
Plasma HDL (mmol/L)	0.91 ± 0.42	> 1.0
Serum creatinine (mmol/L)	85 (67–127)	45–90
Urine ACR (mg/mmol)	21 (3.1–142.8)	< 3.5
Serum Albumin (g/L)	36.2 ± 6.1	34–45
Serum GGT (U/L)	45 (27–93)	< 43
Serum ALP (U/L)	113.5 (85–151)	30–110

Data are presented as count (%) or *SD* standard deviation, *HbA1c* glycated haemoglobin, *LDL* low density lipoprotein, *HDL* high density lipoprotein, *ACR* albumin creatinine ratio, *GGT* Gamma-glutamyl transferase, *ALP* Alkaline phosphatase

The majority (89.6%) of the ulcers were Wagner grade 1–2 and were present on the plantar surface of forefoot (74.9%). The median area of the ulcer was 2.0 cm^2^ (0.8–5.0). Almost half of the patients (49.5%) presented to MDFC within 4 weeks of onset of ulcer, while a small number (6.6%) had long-standing ulcers for more than a year. The most common causes of DFU were trauma (45.0%) and infection (29.4%). Infective causes included preceding cellulitis, an infected fissure, or an infected callus. Up to 7.0% of subjects had traumatic DFU due to barefoot walking and burns. Methicillin-resistant *Staphylococcus aureus* (1.4%) and non-multi-resistant oxacillin-resistant *Staphylococcus aureus* (8.8%) were uncommon in this population.

In general, metabolic control was poor, with an average HbA1c of 9.1 ± 2.5%, LDL of 2.2 ± 0.92 mmol/L, and HDL of 0.91 ± 0.42 mmol/L. Mean haemoglobin was 120.0 ± 20.9 g/L, with 33.3% having levels < 110 g/L, suggesting anaemia of chronic disease and/or malnutrition. WBC elevation of > 11 × 10^9^/L was present in 26.0%, and CRP > 50 mg/L was seen in 20.2%, indicating severe infection. About a half of subjects (51.3%) required surgical debridement of their DFU. Adjunct hyperbaric therapy was required for a quarter (25.5%). A third of subjects (34.3%) were treated with total contact cast, and half (51.3%) underwent amputation over the study duration. A total of 436 amputations occurred during this study period, with an average of 1.75 ± 1.01 amputations per patient. There were 99 major amputations and 337 minor amputations.

**Fig. 1:**
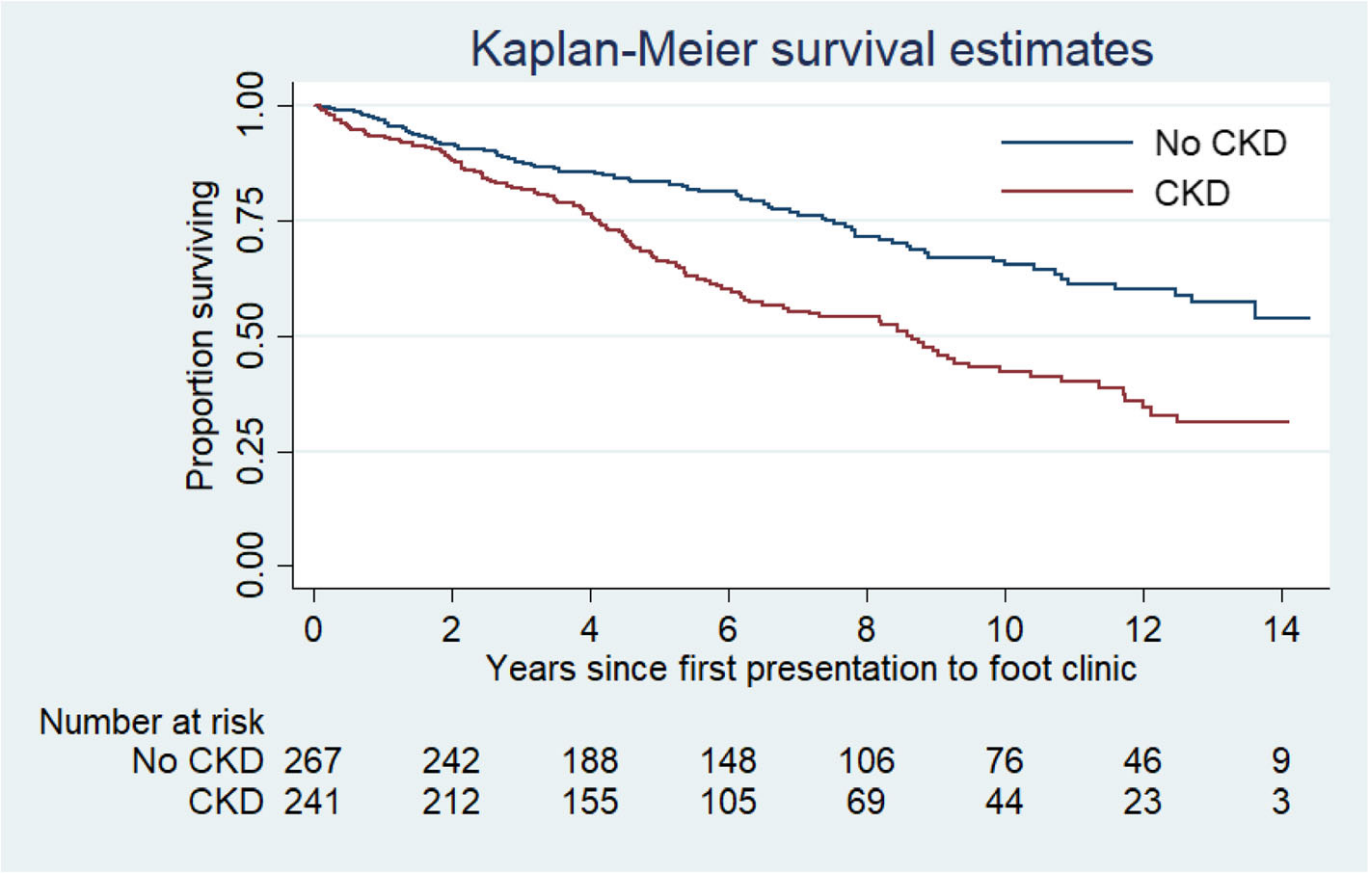
Survival analysis of 513 patients with diabetes foot ulcer in relation to Chronic Kidney Disease (CKD), excluding 5 patients who are renal transplant recipients. The difference between the curves was significant (*p* < 0.0001)

There were 199 deaths recorded during the study period. The 5-year-mortality rate was 24.6%, and the 10-year-mortality rate was 45.4%. In univariate analysis, the main contributors to increased mortality were: age; ethnicity (Indigenous); past DFU; non-compliance; PVD; CKD; macrovascular disease; low haemoglobin; HbA1c; increased urine ACR; and low plasma albumin (Table 2). Gender, remoteness, diabetes duration, ulcer duration, ulcer site, ulcer area, Wagner Grade, amputation, hypertension, smoking, alcohol or other drug abuse, neuropathy, retinopathy, inflammation markers, lipid levels, gamma-glutamyl trans-peptidase, and insulin therapy were not associated with mortality (data not shown). In the final model of multivariate analysis, only age [HR 1.04, 95% CI (1.02–1.05)], CKD [HR 1.22, 95% CI (1.11–1.33)], and plasma albumin [HR 0.96, 95% CI (0.94–0.99)] remained significant. Figure 1 shows the Kaplan-Meier curve for the mortality rate related to CKD.

**Table 2:** Independent associations with all-cause mortality in univariate and multivariate analysis in patients with diabetes foot ulcers

Parameter	Univariate HR	Multivariate HR (Model 1)	Multivariate HR (Model 2)
Age	1.03^***^ [1.02,1.04]	1.12^**^ [1.03,1.20]	1.04^***^ [1.02,1.06]
Ethnicity (Indigenous)	1.45^**^ [1.10,1.92]	0.65 [0.097,4.42]	
Past ulcer	1.43^*^ [1.06,1.92]	2.70 [0.36,20.34]	
Peripheral vascular disease	2.04^***^ [1.54,2.71]	2.41 [0.47,12.41]	
Retinopathy	1.32 [0.97,1.78]	5.13 [0.54,48.97]	
Macrovascular disease	1.78^***^ [1.34,2.36]	12.11^*^ [1.65,88.66]	1.36 [0.92,2.01]
Chronic Kidney Disease	1.24^***^ [1.16,1.33]	1.63^*^ [1.03,2.60]	1.22^***^ [1.11,1.34]
Blood HbA1c	0.92^*^ [0.85,0.99]	1.24 [0.98,1.5]	1.00 [0.92,1.08]
Blood Haemoglobin	0.99^***^ [0.98,0.99]	0.95^*^ [0.90,0.99]	0.99 [0.98,1.00]
Serum Albumin	0.97^**^ [0.95,0.99]	1.31^*^ [1.06,1.63]	0.97^*^ [0.94,0.995]
Serum Alkaline Phosphatase	1.004^***^ [1.002,1.005]	1.007 [0.99,1.02]	
Urine Albumin Creatinine Ratio	1.002^***^ [1.001,1.002]	1.00 [0.999,1.007]	
Non-compliance	2.038^**^ [1.276,3.255]	4.43 [0.33,58.51]	

Hazard Ratio; 95% confidence intervals in brackets; ^*^ *P* < 0.05, ^**^ *P* < 0.01, ^***^ *P* < 0.001; Model 1 included variables with *P* < 0.01 in univariate analysis. Model 2 included variables with *P* < 0.01 in Model 1. Gender, remoteness, diabetes duration, ulcer duration, ulcer site, ulcer area, Wagner Grade, amputation, hypertension, smoking, alcohol or other drug abuse, neuropathy, retinopathy, inflammation markers, lipid levels, gamma-glutamyl trans-peptidase and insulin therapy were not associated with mortality

Table 3 lists the causes of death in this cohort. The most common causes of death were CKD (24.6%), cardiovascular events (19.6%), sepsis (15.6%), respiratory failure (10.0%), malignancy (9.5%) and multi-organ failure (5.0%).

**Table 3:** Cause-specific mortality in 199 patients with diabetic foot ulcers

Cause of death	Total cohort	Place of death
All causes combined	199 (100%)	104 hospital 89 home 6 palliative care
Chronic kidney disease	49 (24.6%)	18 hospital 31 home
Cardiovascular disease	39 (19.6%)	25 hospital 12 home 2 residential care
Sepsis	31 (15.6%)	20 hospital 9 home 2 interstate
Respiratory failure	20 (10%)	15 hospital 3 home 2 residential care
Multi-organ failure	10 (5%)	8 hospital 2 home
Other causes^a^	3 (1.5%)	3 home
Unknown	28 (14.1%)	2 hospital 22 home

^a^Other causes: 1 dementia, 1 gastrointestinal infection and 1 brain abscess

## Discussion

In patients with diabetes, DFU are being increasingly recognised as a marker for high mortality [13–17]. Studies from all over the world report that half of all patients who develop DFU die within 5 years [5]. Our study is the first Australian study conducted in a socially and ethnically diverse population to examine the mortality outcomes in patients with DFU. It is also one of the largest studies wordwide. Almost half of our study cohort were Indigenous and 30% were from remote areas. The patients with DFU died at an age of 64.6 years, which was lower than the Australian average age of death of 80.4 years in males and 84.5 years in females [18].

The 5 year mortality rate in our cohort was 24.6%. Previous studies have reported higher mortality rates of 40 to 51.7% [6, 13–17, 19], with the exception of Pinto et al. [20]*,* who showed a 5 year mortality rate of only 13.7%, and Young et al. [19] with 26.8%. The rate shown by Young et al. was the result of introducing a protocol for aggressive cardiovascular risk management in their foot-clinic, which had lowered their mortality rate from 48%. We cannot directly compare the mortality rate reported due to patient and methodological heterogeneity. Our study population was younger and had shorter diabetes duration compared to the other studies, which might explain the lower 5 year mortality rate. However, the 10-year mortality rate in our study was 45.6%, which was similar to the 49% reported by the only other study that reported 10-year mortality [21].

In the present study, the risk factors independently associated with increased mortality were increasing age and CKD, while patients with higher plasma albumin levels had longer life expectancy. It is not surprising that increased age is associated with increased mortality, and this has also been shown in other patient populations [22]. Moreover, patients with DFU have been shown to be older and had longer duration of diabetes than those without DFU [23, 24].

Patients with later stages of CKD and advanced diabetic nephropathy have a greater risk of complications and mortality. The degree of renal impairment correlates strongly with the incidence and prevalence of DFU [25]. Wolf et al. [26] reported that impaired renal function was an independent predictor of all-cause mortality and cardiovascular deaths. Additionally, albuminuria with advanced renal disease was associated with increased risk of limb amputation in patients with DFU. Moreover, Ghanassia et al. [14] demonstrated that CKD was the only independent predictor of mortality in patients with DFU. Similarly, in our cohort with equal contribution from Indigenous and non-Indigenous Australians, CKD remained a significant risk factor for mortality, even after adjusting for other variables. However, urine ACR by itself was no longer a significant factor in the multivariate analysis. CKD may well be a surrogate marker for microvascular damage, which in turn indicates higher risk of neuropathy and vascular insufficiency, both of which are associated with poor wound healing and survival.

In several studies, mortality was predicted by plasma albumin concentration across a broad range of values in populations with and without disease. The estimated increase in the odds of mortality ranges from 24 to 56% for each 2.5 g/L decrement in plasma albumin concentration [27–29]. In our study, low serum albumin remained an independent predictor of mortality, even after adjusting for other covariates. Low serum albumin, together with lower haemoglobin levels, may be markers of malnutrition and/or chronic disease.

The Australian Indigenous population are known to have higher mortality rates compared to their non-Indigenous counterparts. The mortality rate of Australian Indigenous patients was approximately 10-fold higher when compared with Australian non-Indigenous patients (85.4 per 100,000 compared to 8.7 per 100,000) [12]. A global comparison of Indigenous data between New Zealand, Australia, Canada, and the United States, shows the highest mortality rate for Indigenous Australians and New Zealand Maoris [30]. In our study, ethnicity was associated with greater mortality in univariate analysis, but was not significant after adjusting for other co-variates. This indicates that the risk factors and the disease process of DFU drive mortality, rather than ethnicity. Thus by treating the risk factors and preventing the disease, we could potentially close the gap in the mortality between Indigenous and non-Indigenous populations.

Many other studies in patients with DFU found that male gender was a risk factor for increased mortality [13, 21]. However, this was not demonstrated by our study, despite a male predominance in our cohort. The reason why males are at increased risk for foot ulceration is unclear. It has been suggested that men have higher risk for developing neuropathy as they are taller, and women in the reproductive age group have better endothelial function in their micro- and macro-circulation [31].

PVD was another commonly reported predictor of mortality in DFU patients [32]. In our study, PVD also predicted mortality; however, the significance disappeared when adjusted for other factors. It is likely that CKD was such a dominant risk factor in our cohort that the significance of most other risk factors disappeared.

We found that macrovascular disease was associated with mortality in the univariate but not multivariate analysis. Only occasional studies have confirmed an independent association between cardiovascular disease and death in patients with DFU [33]. The likely explanation is that in patients with DFU there are a myriad of non-cardiovascular causes contributing to increased mortality, such as neuropathy, renal impairment, chronic inflammation, and infection [7].

HbA1c has been varyingly reported to have positive or negative association with death in DFU patients. We found that better HbA1c increased mortality, although this was no longer significant in multivariate analysis. It may be speculated that the increased occurrence of CKD, and the consequent decrease in insulin clearance, may have led to lower HbA1c in the patients who died. Moreover, HbA1c is less reliable in CKD. Winkley et al. observed a similar relationship between HbA1c and death [34]. They suggested the association may be related to increased surveillance and medical management, more frequent hospitalisations, and poor appetite in patients who were more unwell and died.

Lower extremity amputation is often an independent predictor of mortality, with age, PVD, CKD, and proximal amputation, increasing the risk of death after amputation [35]. Five-year survival rates for those who had a limb amputation, as compared to a toe amputation, were poor, with mortality rates ranging from 39 to 80% [17]. However, we did not find any significant association with amputation and mortality. This may be because our population was younger, had less diabetes-related foot complications, shorter duration of diabetes, and the majority had only a minor amputation.

Very few studies have looked at the cause-specific mortality in patients with DFU. CVD is the most common cause of death in patients with DM, and a similar trend is expected in patients with DFU. However, the proportion of cardiovascular deaths in patients with DFU has been reported from 19 to 54.8% [23, 36, 37]. In our study, the major cause of death was CKD. However, this finding is comparable to the cause of death from CKD in Australia and the US in the diabetes population generally [35]. This may also reflect the high burden of renal disease in the NT population.

The main strengths of this study were the long follow-up period and the high proportion of Indigenous patients. The inherent limitations of all retrospective studies were also present in our study, particularly ascertainment bias. Also, there could have been a negative selection bias as the patients were recruited from a high-risk foot service at a tertiary centre, and simple superficial ulcers might have been excluded as they would be managed in a primary care setting. Therefore, the results of the study may not be applicable to primary care. We also acknowledge that the cause of death was not ascertained by post-mortem examinations.

## Conclusion

Patients with DFU have high mortality and reduced life expectancy. Age, CKD, and low serum albumin levels are risk factors for mortality. Indigenous status was a risk factor in univariate analysis, but not in multivariate analysis, indicating that other risk factors determine mortality. The presence of a DFU should be seen by health care providers as an alarming signal to premature death, and should be used to initiate intensive risk factor reduction and close follow-up.

## Abbreviations

ACR: Albumin creatinine ratio
ALP: Alkaline Phosphatase
CI: Confidence interval
CKD: Chronic kidney disease
CRP: C-reactive protein
CVD: Cardiovascular disease
DFU: Diabetic foot ulcers (DFU)
DM: Diabetes mellitus
GGT: Gamma-glutamyl transferase
HbA1c: Glycated haemoglobin
HDL: High density lipoprotein
HR: Hazard ratio
IQR: Interquartile range
KDIGO: Kidney Disease: Improving Global Outcomes (KDIGO) study
LDL: Low density lipoprotein
MDFC: Multi-Disciplinary foot clinic
NT: Northern Territory
PVD: Peripheral vascular disease
RDH: Royal Darwin hospital
WBC: White cell count
